# An Energy-Efficient Routing Protocol for Reliable Data Transmission in Wireless Body Area Networks

**DOI:** 10.3390/s19194238

**Published:** 2019-09-29

**Authors:** Yating Qu, Guoqiang Zheng, Honghai Wu, Baofeng Ji, Huahong Ma

**Affiliations:** 1 School of Information Engineering, Henan University of Science and Technology, Luoyang 471023, China; 170317050197@stu.haust.edu.cn (Y.Q.); honghai2018@haust.edu.cn (H.W.); baofengji@haust.edu.cn (B.J.); mhh@haust.edu.cn (H.M.); 2 Henan Key Laboratory for Machinery Design and Transmission System, Henan University of Science and Technology, Luoyang 471003, China

**Keywords:** WBAN, routing protocol, network lifetime, reliable transmission

## Abstract

Wireless body area networks will inevitably bring tremendous convenience to human society in future development, and also enable people to benefit from ubiquitous technological services. However, one of the reasons hindering development is the limited energy of the network nodes. Therefore, the energy consumption in the selection of the next hop must be minimized in multi-hop routing. To solve this problem, this paper proposes an energy efficient routing protocol for reliable data transmission in a wireless body area network. The protocol takes multiple parameters of the network node into account, such as residual energy, transmission efficiency, available bandwidth, and the number of hops to the sink. We construct the maximum benefit function to select the next hop node by normalizing the node parameters, and dynamically select the node with the largest function value as the next hop node. Based on the above work, the proposed method can achieve efficient multi-hop routing transmission of data and improve the reliability of network data transmission. Compared with the priority-based energy-efficient routing algorithm (PERA) and modified new-attempt routing protocol (NEW-ATTEMPT), the simulation results show that the proposed routing protocol uses the maximum benefit function to select the next hop node dynamically, which not only improves the reliability of data transmission, but also significantly improves the energy utilization efficiency of the node and prolongs the network lifetime.

## 1. Introduction

The wireless body area network (WBAN) is a special branch of the wireless sensor network (WSN), and has great potential in the field of medical and health. WBAN can be used for clinical medical monitoring, chronic disease monitoring, and daily monitoring of elderly or special groups, which can rid patients of the shackles of wired monitoring and allow them to benefit from ubiquitous medical services at anytime and anywhere [1,2]. Therefore, the development of WBAN is attracting more and more attention, becoming one of the hot spots of current and future research [3].

**Figure 1:**
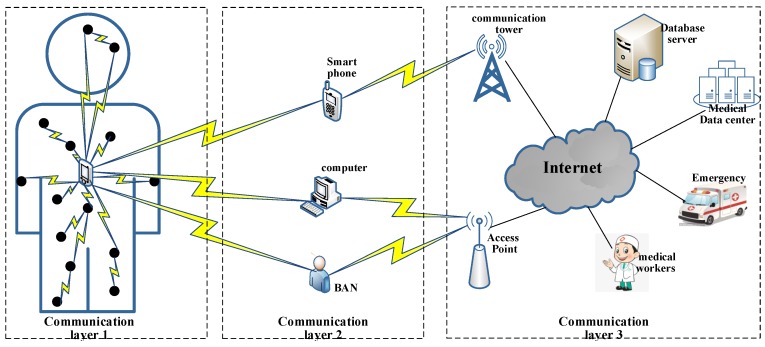
Architecture of wireless body area network communication.

WBAN is a body-wide network whose basic architecture can be divided into three layers [4], as shown in Figure 1. Tier-1 is composed of sensors attached to the body surface or implanted into the body. Its function is to collect and transmit various physiological information about the human body. Tier-2 is composed of smart phones, personal computers, or other intelligent electronic devices. The information sent by sensors is forwarded to the terminal data center by a wireless mode. In tier-3 of WBAN, the terminal data center is mainly composed of remote servers providing various applications. Its function is to collate and analyze the received data, and thereby provide a dynamic response. Emergency transmissions and alarms are performed when sensor nodes collect abnormal data, which speeds up emergency handling and rescue.

One of the difficulties in the design of a wireless body area network is energy limitation [5], because the nodes are powered by micro-batteries, which have limited energy and are difficult to replace. Moreover, since the body is a special transmission medium, human tissue will also absorb a part of the energy [6,7], which is generally defined as the specific absorption rate (the electromagnetic radiation energy absorbed by human tissue per unit time). When the electromagnetic energy accumulated in the human body exceeds a certain value, it will also cause certain damage to human tissue. Therefore, the energy consumption problem this poses will be a research direction in the future. In addition, the deployment of nodes will also affect the energy consumption; frequent use of a node will cause these nodes to prematurely consume their own energy and quit the network [8]. To solve these problems, we can consider the two directions of open source and throttling. The open source method is a variety of wireless energy collection technologies [9,10,11], and the throttling method is a variety of energy-efficiency design. Designing the reasonable routing is one of the methods to improve energy efficiency. Routing is responsible for establishing the path in the network, the design principles of routing are stability and energy efficiency. How to improve the energy efficiency, prolong the network lifetime and avoid network splitting has become an important issue in research of routing protocols for wireless body area networks [12].

Based on the above analysis, this paper proposes an energy-efficient routing protocol for reliable data transmission in WBAN, the protocol not only ensures reliable and efficient routing transmission of data, but also balances the energy consumption of the network and prolongs the network lifetime. The specific contributions are as follows:

(1) A maximum benefit function is constructed to dynamically select the next hop node with the good state. The function takes multiple parameters into account, such as the remaining energy, transmission efficiency, available bandwidth and hops from the sink, which can achieve reliable multi-hop data transmission.
(2) Based on the different priority data of WBAN, we dynamically adjust the weight of the maximum benefit function to achieve timely and reliable transmission of emergency data and also satisfy the Qos requirements of periodic data.
(3) A simulation experiment platform was established to compare the proposed protocol with the PERA and NEW-ATTEMPT routing protocols. The experiment shows that the proposed protocol has significant advantages in prolonging network lifetime and reliable data transmission.

Furthermore, the related work is introduced in Section 2; Section 3 presents the system mode; Section 4 gives a detailed description of the protocol proposed; and the experimental results of our proposed protocol are provided in Section 5. Finally, we summarize this paper in Section 6.

## 2. Related Works

More and more studies have proved that the two-hop or multi-hop method is more suitable for WBAN. It can balance network energy consumption and improve energy efficiency. Therefore, the selection of next-hop node is the hotspot of current research in multi-hop routing [13]. Moid et al. [14] proposed a routing protocol, which considers the residual energy of the nodes for the selection of next-hop nodes, and selects the node with the largest residual energy as the best next hop. This method can balance the energy consumption of the network, but it will cause a large delay or packet loss rate. Ahmed et al. [15] proposed a minimum hop routing protocol to select the best next hop node by the lowest hops from the sink. Although this method can satisfy the Qos demand of low delay, it is easy to cause some fixed nodes to quit the network due to frequent participation in data transmission and excessive energy consumption, thereby affecting the connectivity of the network. The adaptive thermal-aware routing protocol (ATAR) proposed by Jamil et al. [16] is a temperature-based routing protocol, which is designed to overcome the temperature rise issue of implanted bio-medical sensors nodes. This protocol is based on Multi-Ring routing approach to find an alternative route in the case of increasing temperature. Using the retreat strategy to avoid the node with high temperature will cause a large delay. Maintaining ring information, temperature and hops in the network also leads to increased overhead, which is not a good choice for a volume network with limited resources.

A trust and thermal aware routing protocol ( TTRP) was proposed by Bhangwar et al. [17]. This protocol considers both the temperature and trust parameters of the node when selecting the best next hop to ensure the reliability communication between nodes and the safety of human body, but the energy consumption of nodes is not considered. Khann et al. [18] designed a multi-hop routing protocol, the best next hop node selected has the characteristics of more residual energy and closer to the sink. This method considers the residual energy of the node, but fails to achieve the goal of energy efficiency. Its disadvantage is to accelerate the energy consumption of the node in the network center, which leads to the early death of the node and affects the connectivity of the network.

Smita et al. [19] proposed a modified new-attempt routing protocol, which constructs a cost function to select the next hop node. The cost function is calculated using the distance among the nodes, residual energy and the rate of the sending data. Therefore, this method meets the Qos of energy efficiency and low delay. Su et al. [20] proposed an enhanced mobility and temperature-aware routing protocol, and this protocol takes three routing metrics: hop count, temperature and link quality to select the best next hop. The disadvantage is that the energy consumption of nodes is not considered. Ghufran et al. [21] proposed a thermal and energy-aware routing protocol, and consider the weighted average of three costs while selecting the routing path: energy consumption, heat dissipation, and link quality. The best next hop node has the characteristics of more residual energy, lower temperature and higher link quality, which can meet several Qos requirements.

An energy-aware link-efficient routing protocol was proposed by Anwar et al. [22], and a multi-parameter cost function is constructed when selecting the best next hop node. The residual energy, link quality, hops and distance to the sink are considered comprehensively, and the weight factors of each parameter are set artificially. Although the protocol meets multiple Qos requirements, it does not consider the priority of data classification and transmission, so it cannot guarantee the timely and reliable transmission of emergency data. Similarly, Sangwan et al. [23] proposed a reliable energy efficient multi-hop routing protocol, and this protocol constructs a cost function which considers the parameters of the residual energy, the distance to the sink, the failure probability and the communication count to realize reliable and efficient data transmission.

Similarly, there are more methods to find the best next hop node by constructing cost function [24,25,26,27,28,29,30]. In multi-hop routing, the selection method of the next hop node is the core of the research, which is related to the comprehensive performance of the network [31]. Through the study of the above routing protocols, there are still some shortcomings in the next hop node selection method. For example, the constructed cost function considers single parameters, and the proposed protocol does not consider data classification and priority processing. Of course, it cannot meet the requirement of multiple Qos in a wireless body area network. Therefore, based on the above background, this paper proposes an energy-efficient routing protocol for reliable data transmission in WBAN. Unlike the current research results, this protocol takes a number of parameters into account and constructs a maximum benefit function by normalization, which is used to dynamically select the next hop node with a good state. It can not only achieve reliable and efficient routing transmission of data, but also improve the energy efficiency of network, and thereby prolong the network lifetime.

Following is a detailed description of the system model, construction of the maximum benefit function, routing process and performance evaluation of the proposed routing protocol.

## 3. System Model

The system models considered in this protocol includes a network model and energy consumption model of nodes. Detailed contents are as follows.

### 3.1. Network Model

**Figure 2:**
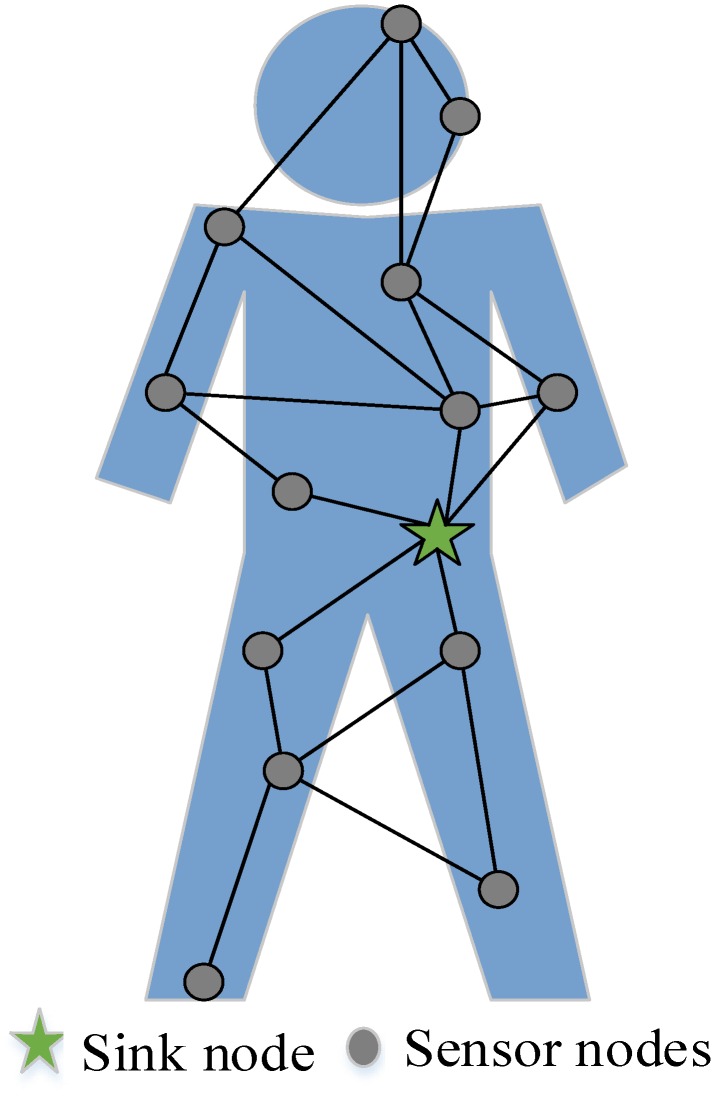
Logical topology of wireless body area network.

As shown in Figure 2, a wireless body area network consists of a sink and multiple sensor nodes. The sink node is composed of smart phones, personal computers or other smart electronic devices. Sensor nodes are all kinds of biosensors, which are powered by micro-batteries. Each node is responsible for collecting one or more kinds of physiological data. At the same time, the collected data is transmitted to the sink node by wireless transmission, and then sent to the medical data center by the sink for comprehensive processing. The data transmission uses one or more hops, and the maximum hops should not exceed three hops.

In practical applications of WBAN, the data generated by nodes are mainly divided into two categories: emergency data and periodic data. Emergency data refers to abnormal data exceeding the normal threshold and query data from users’ active requests, which are generated randomly and have small business. At the same time, the transmission of emergency data requires high real-time reliability. Therefore, emergency data is designed as P1 priority data that needs to be processed first. Periodic data refers to continuous or discontinuous periodic data generated by nodes. This kind of data has a large amount of traffic and is transmitted periodically to sink nodes. Compared with emergency data, the periodic data has low real-time requirements. Therefore, the periodic data is designed as P2 priority data.

Model assumptions:

(1) All sensor nodes are distributed in the corresponding position of the human body according to their different functions. After placement, all sensor nodes have their own ID and their positions remain unchanged.
(2) The initial energy of each node is equal, and all nodes have data fusion function. At the same time, the transmitting power can be dynamically adjusted, and the maximum wireless transmission distance is R.
(3) When the node is sleeping, the wireless module is closed and the low-energy detection module is still working.
(4) According to the actual situation, the energy of the sink node is not considered or assumed to be infinite, and it has strong information-processing ability.

### 3.2. Energy Consumption Model

The energy consumption of nodes mainly comes from data collection, data transmission, data receiving and idle interception. Among them, the energy consumption of data transmission accounts for the main part. Because this paper mainly studies the communication between nodes, only the energy consumption of sending and receiving is considered, while the energy consumption of data collection and idle listening is neglected.

In this paper, the proposed energy consumption model is used in [32]. In this model, the energy consumption for transmitting and receiving k bit data is expressed as follows:

(1) $$E_{\mathrm{rx}}(k)=E_{elec}\times k$$

(2) $$E_{\mathrm{tx}}(k,d)=E_{elec}\times k+E_{amp}\times k\times d^{2}$$

where $E_{rx}(k)$ denotes the energy consumed by receiving k bit data, $E_{tx}(k,d)$ denotes the energy consumed by sending k bit data to the node at distance. $E_{elec}$ and $E_{amp}$ represent the energy consumed by the circuit when the node sends or receives data and the energy consumed by the power amplifier when sending data, respectively.

If the initial energy of node i is $E_{i}^{initial}$, the residual energy is $E_{i}^{res}$, and the energy consumed is $E_{i}^{con}$, then the quantitative relationship shows that:

(3) $$E_{i}^{con}=E_{rx}+E_{tx}$$

(4) $$E_{i}^{res}=E_{i}^{initial}-E_{i}^{con}$$

## 4. Energy-Efficient Routing Protocol for Reliable Data Transmission in WBAN

This paper proposes an energy efficient routing protocol for reliable data transmission in WBAN, which adopts two-hop or multi-hop communication to alleviate the energy consumption caused by direct communication. We also construct a multi-parameter maximum benefit function to dynamically select the next-hop node with good state and dynamically adjusts the weight of the maximum benefit function based on the priority of data. Reliable and efficient data transmission is achieved via dynamic routing adjustment in this paper. Following this, the construction of the maximum benefit function and the routing process are described in detail.

### 4.1. Construction of the Maximum Benefit Function

The maximum benefit function is to normalize the residual energy, transmission efficiency, available bandwidth and hops to the sink, and then sum them up after allocating the weighted coefficients respectively. Therefore, the node with the largest value of the function is the best next hop.

Since the energy of nodes is limited, it is necessary to improve the energy efficiency [33]. This paper firstly considers the residual energy of the nodes, choosing the nodes with more residual energy as the best next hop and thereby can balance the overall energy consumption of the network. Formula (4) is used to calculate the residual energy $E_{i}^{res}$ of the node. The normalized expression is as follows:

(5) $$\gamma_{i1}=\frac{E_{i}^{res}-E_{min-th}}{E_{i}^{initial}-E_{min-th}}$$

where $\gamma_{i1}$ denotes the normalized parameters of the residual energy, $E_{i}^{initial}$ denotes the initial energy of the node i, i.e. the maximum energy of the node, and $E_{\min-th}$ denotes the minimum threshold of the node. When the residual energy of the node is lower than the threshold, only their own data are transmitted.

The transmission efficiency of a node is the ratio of packets forwarded successfully. The more packets forwarded successfully per unit time, the higher the transmission efficiency of the node. Selecting nodes with high transmission efficiency as the best next hop can ensure reliable data transmission between nodes. The expression of the transmission efficiency $\eta_{i}$ is as follows:

(6) $$\eta_{i}=\frac{P_{i}^{success}}{P_{i}^{receive}}$$

where $P_{i}^{receive}$ and $P_{i}^{success}$ represent the number of packets received by the node i and the number of packets successfully forwarded. The transmission efficiency $\eta_{i}$ are normalized as follows:

(7) $$\gamma_{i2}=\frac{\eta_{i}-\eta_{min}}{\eta_{max}-\eta_{min}}$$

where $\gamma_{i2}$ denotes the normalized parameter of transmission efficiency,$\eta_{max}$ and $\eta_{min}$ represent the maximum and minimum transmission efficiency, respectively.

The available bandwidth of a node is the bandwidth currently available. The wider the available bandwidth, the better the data transmission will be. Considering the available bandwidth of nodes can further ensure the reliable transmission of data and improve the performance of the network. The available bandwidth $B_{i}^{av}$ of the node i is normalized as follows:

(8) $$\gamma_{i3}=\frac{B_{i}^{av}-B_{min}}{B_{max}-B_{min}}$$

where $\gamma_{i3}$ denotes the normalized parameters of the available bandwidth of the node, $B_{max}$ and $B_{min}$ denote the maximum bandwidth and minimum bandwidth respectively.

After network initialization, the node knows the hops to the sink. Choosing the appropriate next hop node can reduce transmission delay and ensure real-time communication between nodes. $H_{i}$ denotes the hops of the candidate next hop node to the sink and normalizes it as follows:

(9) $$\gamma_{i4}=\frac{H_{i}}{H_{max}}$$

where $\gamma_{i4}$ denotes the normalized parameter with the hops and $H_{max}$ denotes the maximum hops of the candidate next hop node to the sink.

A simple linear weighted sum of the above four normalized parameters is used to construct a maximum benefit function $M_{i}$, which can evaluate the performance of each candidate next hop node, and then select the best next hop node. The maximum benefit function is expressed as follows:

(10) $$M_{i}=\alpha *\gamma_{i1}+\beta *\gamma_{i2}+\theta *\gamma_{i3}+\lambda *(1-\gamma_{i4})$$

where α,β,θ,λ is the weight factor of each parameter, and α+β+θ+λ=1, which needs to be selected according to experience in practical application.

### 4.2. Routing Process

**Figure 3:**
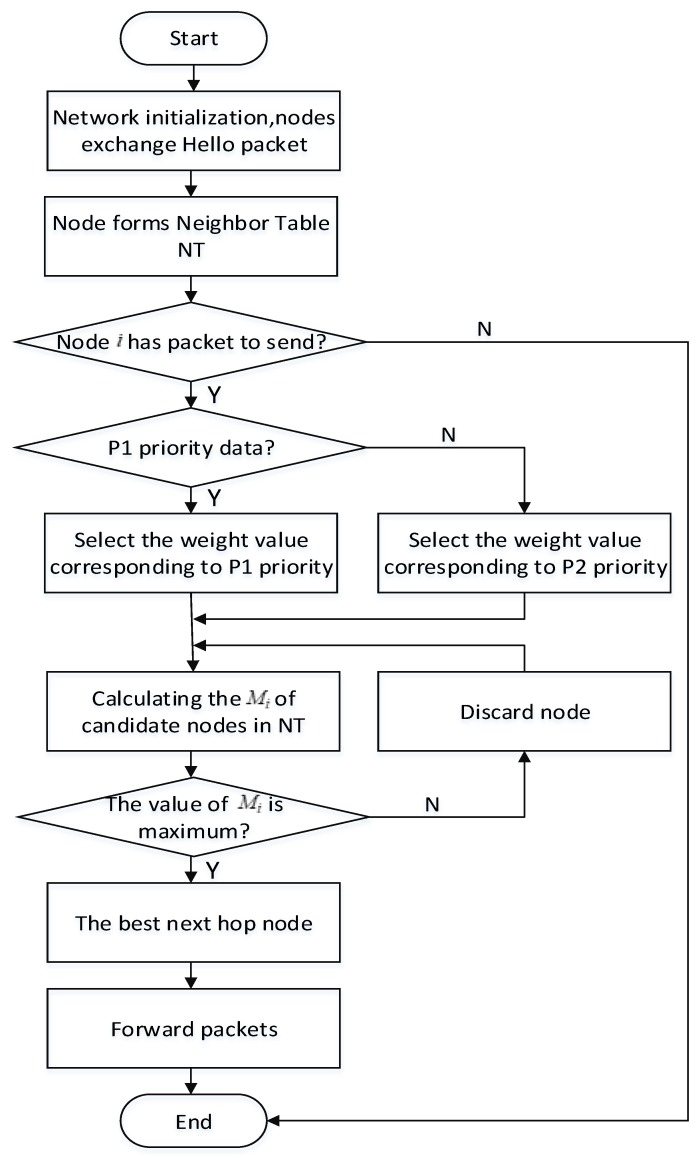
Flow chart of the proposed protocol.

The routing process of the proposed protocol is divided into three stages: initialization stage, the best next hop node selection stage and data forwarding stage. The overall routing process is shown in Figure 3.

#### 4.2.1. Network Initialization

In this phase of the proposed protocol, the sink node broadcasts a Hello message to the whole network, which includes the location of the sink node. After receiving the message, all the nodes update the location of the sink immediately, and then reply with a Hello message to the network. The message includes the nodes’ ID, location, residual energy, transmission efficiency, available bandwidth, hops to sink and so on. At this time, all nodes know the specific location of the sink and its neighbors. After the initialization phase is completed, each node will establish a neighbor nodes information table *NT* (Neighbors Table), and the best next hop will be generated in *NT*.

#### 4.2.2. Next Hop Node Selection Based on Maximum Benefit Function $M_{i}$

The best next hop node is the node with the largest $M_{i}$ value, which satisfies the following formula:

(11) $$\hat{i}=\arg\underset{i}{\max}\left\{M_{i}\right\}=\arg\underset{i}{\max}\left\{\alpha *\gamma_{i1}+\beta *\gamma_{i2}+\theta *\gamma_{i3}+\lambda *(1-\gamma_{i4})\right\},i=1,2,\ldots ,N$$

In the practical application of WBAN, different priority data need to be classified and processed to satisfy the Qos requirements of different data [34]. For P1 priority data, the data are generated randomly and its traffic is small, but the real-time and reliability requirements are extremely high. Because emergency data means abnormal physiological data, which will endanger people’s lives and health, a smaller delay may cause irreversible tragedy. Therefore, it is necessary to increase the weights of parameters such as node transmission efficiency, available bandwidth and hops to the sink. For example, we can set the weights to 0.1, 0.3, 0.4, 0.2 to ensure timely and reliable transmission of emergency data. However, for P2 priority data, we should reduce energy consumption as the premise, and properly increase the weight values of residual energy parameters. For example, we can set the weights to 0.5, 0.2, 0.2, 0.1, which can balance the energy consumption of nodes and satisfy the reliable data transmission. The algorithm for dynamically select weight values for different priority data is presented in Algorithm 1.

**Algorithm 1:** Dynamically select weight values for different priority data**Input:** node i;**Output:** the weight value for the data of node i;Process:1: if node i has packet to send then2:  Judge the priority of the packet;3: else
4:  end 5: end if 6:  if the packet belongs to P1 priority then7:   Select the weight value for P1 priority;8:  else9:   Select the weight value for P2 priority;10:  end if11: Retune the weight value for the data of node i;

The best next hop node selected has more residual energy, higher transmission efficiency, wider available bandwidth and smaller hops. It can not only satisfy the low delay and reliable communication between nodes, but also balance the energy consumption of nodes and prolong the network lifetime. In addition, the protocol stipulates that the total number of hops in multi-hop routing should not exceed three hops. The algorithm for the best next hop selection is presented in Algorithm 2.

**Algorithm 2:** The best next hop selection procedure**Input:***S*: source node, *Sink:* coordinator, $M_{i}$: cost function of node i, *NT*: neighbor table; **Output:***N-best (*i*)*: the best next hop node for iProcess:1: Start2: for *S* has packets to transmit with *Sink* then3:  Select the best next hop from *NT;*4:   for each record in *NT* to5:    Calculate $M_{i}=\alpha *\gamma_{i1}+\beta *\gamma_{i2}+\theta *\gamma_{i3}+\lambda *(1-\gamma_{i4})$;6:    List the set of L←$M_{i}$ value of each record in *NT;*7:     *N-best (*i*)*←max {L}8:    end for9: end for10: end

Because the energy of network nodes is limited, this protocol sets a minimum energy threshold $E_{min-th}$ for each node. When the residual energy of the network node is greater than that threshold, it is allowed to enter the NT as a candidate for the next hop. When the residual energy of the network node is lower than that threshold, it will exit the NT and no longer acts as the next hop. At this time, the node will not forward the information of other nodes but only transmit its own packets. Setting the $E_{min-th}$ can not only avoid a node acting as a relay many times, but also balance the energy consumption of the network, improve the energy utilization efficiency, and prolong the network lifetime.

#### 4.2.3. Data Forwarding

When the best next hop node is selected, data is transmitted. The source node sends the data to the next hop node, and then repeats the above steps until the data is sent to the sink. When a node is selected as the next hop by two other nodes at the same time, this node first forwards P1 priority data to ensure timely transmission of emergency data. Assuming that the initial energy of the node is the same, when the residual energy of a node is less than $E_{min-th}$, the node only transmits its own data.

## 5. Experimental Results

This protocol is simulated on MATLAB platform. The node deployment is shown in Figure 2. Assuming that there are 10 sensor nodes and 1 sink node, the location of the node will not change after deployment. Detailed parameter values are shown in Table 1. In order to verify the performance of the proposed protocol, this paper compares it with the minimum hop routing protocol PERA [15] and the mulit-hop routing NEW-ATTEMPT [19], since the two protocols are similar to our proposed routing. There are many evaluation criteria defined for performance evaluation. We selected four criteria: network lifetime, throughput, residual energy and reliability transmission of emergency data.

**Figure 4:**
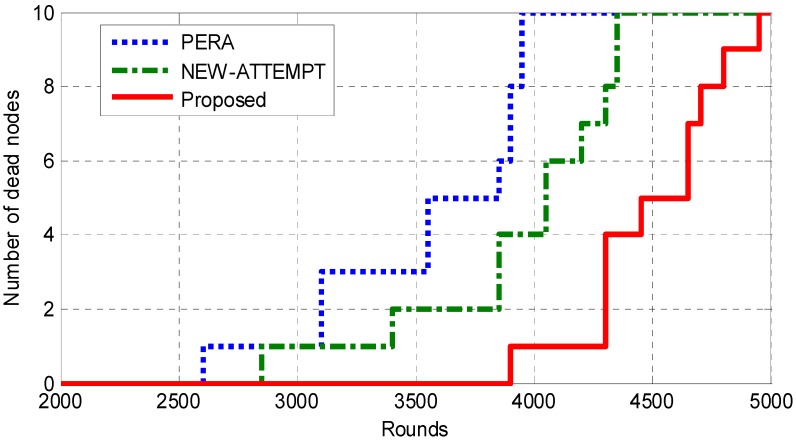
Analysis of network lifetime.

**Figure 5:**
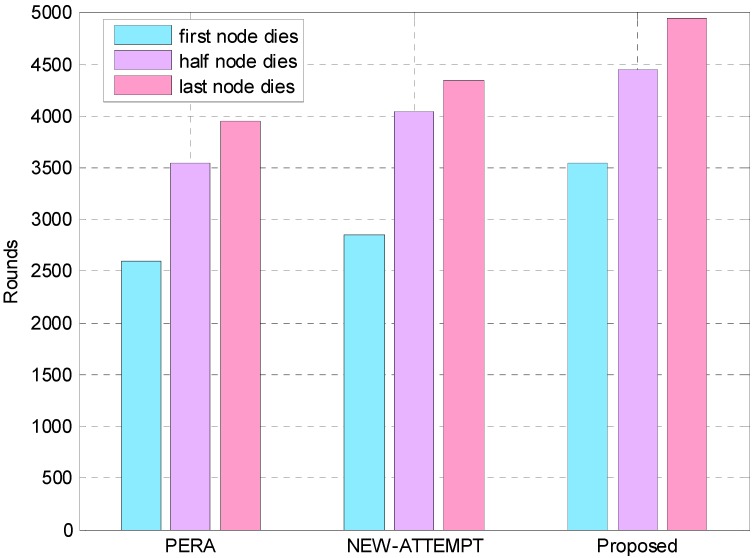
Analysis of network lifetime.

The network lifetime is defined as time taken in rounds from the beginning of network up to the last node dies [35]. The time duration from the start of the network up to the first node runs out of battery is the stability period of the network. Figure 4 and Figure 5 depict the network lifetime comparison of the proposed routing protocol with PERA and NEW-ATTEMPT. We can see that the proposed protocol is superior to the other two protocols in terms of network lifetime by analyzing the experimental data. The PERA protocol selects the node with the smallest hops to sink as the best next hop. The advantage of the PERA protocol is that it can effectively reduce the transmission delay. However, this protocol creates additional energy losses due to the large transmission distance. Based on the above situation, the PERA protocol is not the best choice in WBAN with limited energy resources. Moreover, the NEW-ATTEMPT protocol constructs a cost function to select the best next hop node. The function takes some network parameters into account, such as the remaining energy of the node, the average data rate, and the distance from the sink node. However, the cost function does not fully consider the reliability transmission between nodes. Therefore, the probability of data transmission failure and retransmission is large. This will bring more additional energy consumption, and thereby affect the network lifetime. The maximum benefit function is constructed in this paper, and we consider multiple parameters to improve the efficiency of energy utilization and prolong the network lifetime. Figure 5 shows that the first dead node appears after 3548 rounds in the proposed protocol, while PERA and NEW-ATTEMPT protocols appear in the first dead node after 2604 and 2852 rounds respectively. The network stability period of this protocol is 1.36 times and 1.24 times as much as PERA and NEW-ATTEMPT, respectively. This shows that the proposed protocol improves the energy efficiency and prolongs the network lifetime.

**Figure 6:**
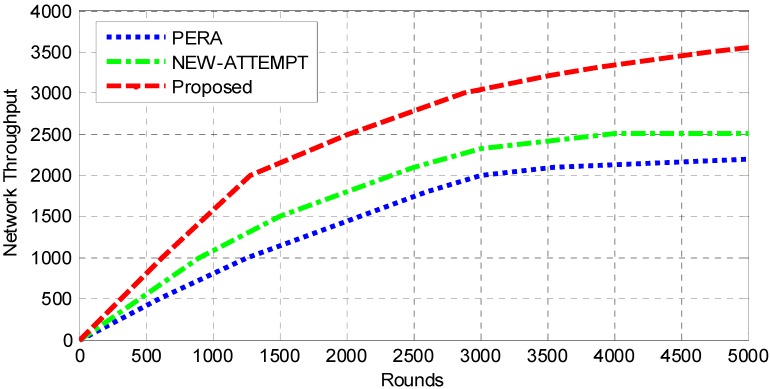
Analysis of network throughput.

The network throughput refers to the successful data transmitted to the destination [36]. Figure 6 shows that the throughput of the proposed protocol in contrast to PERA and NEW-ATTEMPT. It can be seen that the line trend of this protocol is higher than the other two protocols. The throughput achieved by the proposed protocol, PERA and NEW-ATTEMPT are nearly 2200, 2510 and 3550 respectively. The higher throughput achieved by this protocol is to construct a maximum benefit function, which takes multiple parameters into account. Considering the transmission efficiency and available bandwidth of nodes, reliable transmission between nodes can be guaranteed. Therefore, the probability of successful data transmission per unit time is higher, and the throughput is also increased. The lower throughput of the PERA and NEW-ATTEMPT protocols is because neither of them considers reliable communication between nodes. Therefore, the probability of data being successfully transmitted to the sink in a unit time is low, which greatly affects the throughput of the network. Moreover, the network lifetime and network stability period of the other two protocols are also lower than the proposed protocol. Therefore, the throughput is also low.

**Figure 7:**
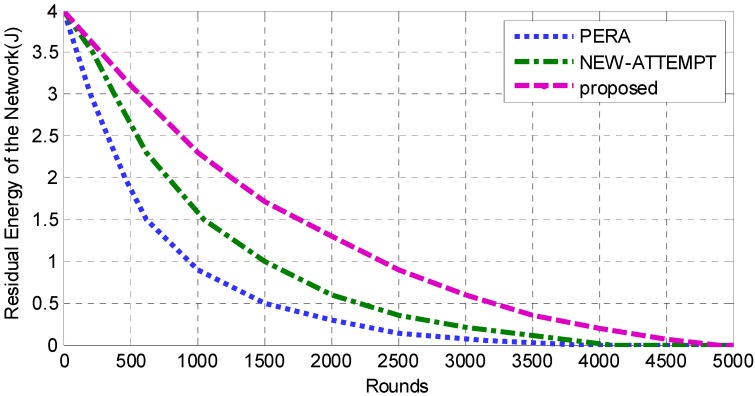
Analysis of energy consumption.

In order to analyze the energy efficiency of routing protocols, it is necessary to detect the energy utilization of each round. Figure 7 shows the energy consumption of the proposed protocol against the other two protocols. The result presents that the energy efficiency of this protocol is higher than PERA and NEW-ATTEMPT. The curve of the PERA protocol declines faster, because its routing method is too simple. Due to the large communication distance, the routing method with the minimum hop count will cause a low probability of successful data transmission. Therefore, most energy of the PERA protocol is used for data retransmission, and the energy utilization efficiency of this protocol is low. Similarly, the NEW-ATTEMPT protocol does not consider reliability parameters when constructing the cost function, which also results in a large transmission failure rate, and most energy of the NEW-ATTEMPT protocol is also used for data retransmission. However, the cost function takes the residual energy parameters into account. Compared with the PERA protocol, the protocol is slightly higher in energy utilization efficiency. The proposed protocol in this paper constructs a maximum benefit function to synthetically evaluate the parameters of the nodes to select the nodes with a relatively good state as the next hop, and improve the energy efficiency. Therefore, the energy efficiency curve is relatively gentle.

**Figure 8:**
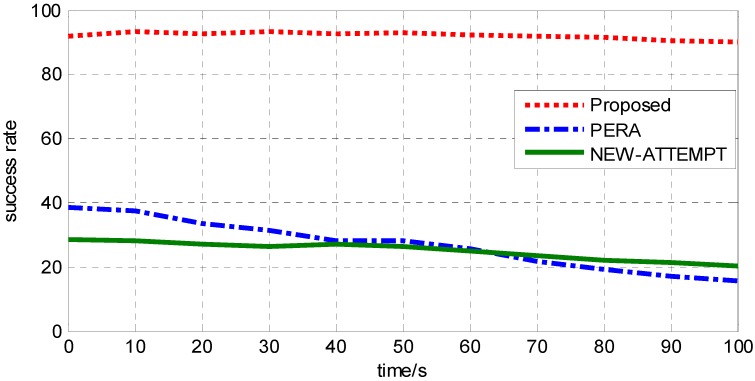
Analysis of the reliable transmission of P1 priority data.

In order to show that this protocol supports reliable transmission of high-priority data, it is necessary to track and record the data of P1 priority. Figure 8 is a comparative analysis of the three protocols on reliability transmission of P1 priority data. From the trend of the lines in the figure, we can see that the successful transmission probability of P1 priority data in this protocol is more than 90%. This is because a maximum benefit function is constructed, and the benefit function sets different weight values for P1 priority data, which can better ensure the reliability of high-priority data transmission. The PERA protocol considers the priority design of data, but the route with minimum hops cannot guarantee the reliable transmission of emergency data, so its corresponding line trend is declining. The NEW-ATTEMPT protocol does not consider the priority of data, so it cannot guarantee the reliable transmission of high-priority data. Its line trend is gentle but low and only stable at about 23.5%.

## 6. Conclusions

In this paper, an energy-efficient routing protocol for reliable data transmission in WBAN is proposed. The protocol dynamically selects the next hop node with good state by constructing a maximum benefit function, which takes multiple parameters into account, such as the residual energy, transmission efficiency, available bandwidth and the hops to sink. At the same time, it dynamically adjusts the weights of each parameter based on the data priority. It can not only ensure reliable and efficient routing transmission of different priority data, but also balance the energy consumption of the network, improve the energy utilization efficiency of nodes, and ultimately prolong the network lifetime. Compared with the minimum hop-routing protocol PERA and the multi-hop routing protocol NEW-ATTEMPT, the proposed protocol performs well in terms of network throughput, network lifetime, energy efficiency and reliable transmission of emergency data.

From the perspective of future work, we plan to further optimize the parameter selection in the maximum benefit function to make it more reasonable and perfect. In addition, we will use a better algorithm or simulation to determine the specific value of the weight, and set reasonable weight values for different priority data to satisfy the Qos requirements of different data, thus achieving better network performance.

## Figures and Tables

**Table 1:** Simulation parameters.

Parameter	Value
Number of nodes	10
Number of Sink	1
Initial energy	0.5 J
Size of a packet	50 bits
$E_{TX-}{}_{elec}$	16.7 nJ/bit
$E_{RX-}{}_{elec}$	36.1 nJ/bit
$E_{amp}$	1.97 nJ/bit/mn
